# Synergistic Sono-Enhanced Photocatalytic Degradation of Antibiotics: Unlocking the Potential of Heterojunctions and Piezoactive Composite Membranes

**DOI:** 10.3390/polym18131643

**Published:** 2026-07-01

**Authors:** Samar Ben Atig, Bruna F. Gonçalves, Moufida Chaari, Samia Dhahri, Hugo Salazar, Fathi Jomni, Senentxu Lanceros-Mendez

**Affiliations:** 1 Laboratory of Materials Organization and Properties (LMOP), Faculty of Sciences of Tunis, University of Tunis El Manar, LR99ES17, Tunis 2092, Tunisia; samar.benatig@etudiant-fst.utm.tn (S.B.A.); samia.dhahri@esstst.utm.tn (S.D.); fathi.jomni@fst.utm.tn (F.J.); 2 BCMaterials, Basque Center for Materials, Applications and Nanostructures, UPV/EHU Science Park, 48940 Leioa, Spain; bruna.ferreira@bcmaterials.net (B.F.G.); senentxu.lanceros@bcmaterials.net (S.L.-M.); 3 Faculty of Science and Technology, University of the Basque Country, 48940 Leioa, Spain; 4 Laboratory of Microbial and Enzymatic Biotechnologies and Biomolecules (LBMEB), Center of Biotechnology of Sfax, University of Sfax, Sfax 3029, Tunisia; moufida.chaari@cbs.rnrt.tn; 5 Higher School of Health Sciences and Techniques of Tunis, University of Tunis El Manar, Tunis 2092, Tunisia; 6 IKERBASQUE, Basque Foundation for Science, 48009 Bilbao, Spain; 7 Laboratory of Physics for Materials and Emergent Technologies (LapMET), Physics Centre of Minho and Porto Universities (CF-UM-UP), University of Minho, 4710-057 Braga, Portugal

**Keywords:** ciprofloxacin, composite membrane, heterojunction, multifunctional, sono-enhanced photocatalysis

## Abstract

The remediation of contaminants of emerging concern (CECs) requires innovative, high-efficiency, and sustainable technologies. Here, we investigate active polymeric membranes incorporating TiO_2_/ZnO heterojunctions for synergistic sono-enhanced photocatalytic water treatment under both UV and visible-light irradiation. TiO_2_/ZnO composites were synthesized and characterized, confirming the formation of type II heterojunctions with tailored optical properties for sunlight-driven photocatalysis. The catalysts were integrated into poly(vinylidene fluoride-co-hexafluoropropylene) (PVDF-HFP) and poly(vinylidene fluoride-co-trifluoroethylene) (PVDF-TrFE) matrixes using electrospinning (ES) and thermally induced phase separation (TIPS). ES membranes, specifically the ZnO-rich heterojunction within a PVDF-TrFE matrix (3T-7Z@TrFE ES), achieved the highest performance toward ciprofloxacin (CIP) degradation, reaching 71 and 57% under UV and visible light, respectively. The hybridization of the method by coupling ultrasound induced significant synergistic effects, with relative enhancement factors up to 1.38. Furthermore, the sono-enhanced photocatalytic pathway shifted the degradation mechanism towards the early fragmentation of the harmful piperazine ring, yielding a more sustainable degradation process. In addition, the composite membranes showed selective antibacterial activity against *S. aureus*, making this a multifunctional platform able not only to degrade CECs but also to mitigate membrane fouling. Overall, this work demonstrates the potential of tailored heterojunctions and composite membranes as sustainable platforms for the remediation of recalcitrant CECs in water, highlighting the synergy between photoactivity, piezoelectricity, and mechanistic control.

## 1. Introduction

Water is essential for life, playing a central role in ecosystems, agriculture, and human health. However, with growing global population and industrial activity, the demand for clean water is increasing, while water resources are being increasingly contaminated, creating a serious environmental challenge [1]. Contaminants such as organic dyes and pesticides, pharmaceuticals, and heavy metals are now frequently detected in natural water sources, posing risks to aquatic life and public health [2].

Contaminants of emerging concern (CECs), particularly antibiotics, present a particularly relevant challenge due to their biological activity, environmental persistence, and resistance to conventional treatment technologies. Global consumption of antibiotics is expected to rise by 67% between 2010 and 2030 [3]. After consumption, around 95% of these antibiotics are excreted in their unmetabolized form through urine and feces, thus entering water cycle. Due to their rising consumption, antimicrobial resistance is increasing and it is estimated to cause 10 million deaths a year in 2050 [4]. A notable example is ciprofloxacin (CIP), a widely used fluoroquinolone antibiotic that is often detected in surface water, groundwater, and wastewater effluents [5]. Its high stability and low biodegradability not only hinder its removal by standard treatments but also contribute to microbial resistance and toxicity to non-target species [6].

To address this, techniques such as filtration, coagulation, adsorption, or biological processes have been studied. However, all of them present limited efficiencies for recalcitrant CECs, leading to the pursuit of new efficient, practical, and sustainable solutions [7]. Advanced oxidation processes (AOPs) have emerged as a promising class of technologies for the destruction of persistent CECs [8]. Within this category, photocatalysis is notable for using light to generate reactive oxygen species (ROS). In this process, light absorption activates a semiconductor material, generating electron–hole (e^−^/h^+^) pairs that drive a cascade of redox reactions leading to the oxidative degradation of contaminants [9]. However, key limitations such as rapid charge recombination and a narrow ultraviolet (UV) light absorption range have led to the development of hybrid strategies [10]. Sonocatalysis, a technique employing ultrasonic waves, boosts CECs’ degradation through the rapid creation and collapse of cavitation bubbles, generating extreme localized heating and high-pressure conditions in water (5000 K, 1000 atm) [11]. With these, generated bubbles split water and oxygen, generating hydroxyl (^•^OH) and superoxide (^•^O_2_^−^) reactive oxygen species (ROS), playing a critical role in CEC degradation [12]. An interesting approach is sono-enhanced photocatalysis, which synergistically combines light irradiation and ultrasonic waves to enhance ROS generation, catalyst dispersion, and mass transfer [13]. This synergy can improve efficiency even in complex or low-light conditions, well-matching with real-water decontamination conditions.

Among a wide plethora of photocatalytic materials, titanium dioxide (TiO_2_) stands out due to its strong oxidative potential, stability, and low toxicity. Yet, its application is mainly limited to UV light due to its wide bandgap, as well as the rapid charge carrier recombination, hindering its practical application. To improve its photocatalytic activity, different strategies have been explored, including noble metal doping or the development of heterojunctions [10]. Zinc oxide (ZnO) is often combined with TiO_2_ to form heterojunctions able to improve charge separation and ROS generation due to their favorable band alignment, a design which facilitates the migration of electrons and holes in opposite directions, thereby reducing recombination [14]. *Charafi, S.* et al. developed a ZnO-TiO_2_ heterojunction able to degrade amoxicillin under UV–visible radiation, achieving 94% of efficiency, and demonstrated the broader applicability under visible light [15]; similarly, a ZnO-TiO_2_ heterojunction was tested for the degradation of tetracycline under visible light, demonstrating that the formation of these heterostructures offers a facile route to improve catalytic degradation [16]. This architecture also expands the active surface area and introduces piezoelectric properties inherent to ZnO, resulting in strong sonocatalytic activity [17]. This dual-activation allows for the efficient degradation of recalcitrant CECs under both solar and acoustic energy. However, a common challenge with powdered photocatalysts is their tendency to aggregate, difficulty in recovery, and risk of secondary contamination.

To overcome this, membrane-based technologies are being explored, integrating the catalysts into a polymeric substrate [18]. Polymers like polyvinylidene fluoride (PVDF) and copolymers are particularly suitable due to their excellent chemical resistance, mechanical strength, and thermal stability, as well as high dielectric constant, and piezoelectric properties [19,20]. In addition, they allow for simple processability into different morphologies. Thermal-induced phase separation (TIPS) and electrospinning (ES) are among the techniques enabling the preparation of PVDF-based membranes with different morphologies: TIPS allows for the processing of interconnected porous structures, while ES leads to porous fibrous networks with a large surface area [21]. As their morphology, structure, and mechanical properties can be controlled, PVDF membranes can be incorporated with functional catalysts tailored to optimize contaminant–catalyst interaction, enhancing the decontamination performance [22]. More importantly, PVDF-based polymers exhibit piezoelectric properties, meaning they can generate electrical charge under mechanical stress, such as that provided by ultrasonic waves [23].

In this context, this study investigates the key role of the PVDF matrix in a hybrid sono-enhanced photocatalytic system. It goes beyond the conventional view of the polymer as a passive support and evaluates it as an active contributor on catalytic reactions [24]. To this end, ZnO-TiO_2_ heterojunctions have been synthesized and integrated into two PVDF copolymers: poly(vinylidene fluoride-co-hexafluoropropylene) (PVDF-HFP) and poly(vinylidene fluoride-co-trifluoroethylene) (PVDF-TrFE), both obtained by ES and TIPS in the form of membranes. The goal is to unravel the relationship between catalyst composition, polymer matrix, and membrane architecture, and to demonstrate how this interplay unlocks a synergistic CIP degradation, establishing a new design principle for advanced water treatment hybrid membranes.

## 2. Experimental Setup

### 2.1. Chemicals

PVDF-HFP (SOLEF^®^ 21,216/1001, 600,000 g/mol, 12 wt. % HFP) and PVDF-TrFE (55/45, Piezotech^®^ FC45) were purchased from Piezotech (Pierre-Benite Cedex, France). N,N-Dimethylformamide (DMF, ≥99%) and ciprofloxacin (≥98%, C_17_H_18_FN_3_O_3_) were supplied by Sigma-Aldrich (Saint Louis, MO, USA). Titanium dioxide (TiO_2_ P25 Aeroxide), with a specific surface area of 35–65 m^2^/g, was provided by Evonik Industries AG (Essen, Germany). Zinc oxide (ZnO, 30 nm, 99%) was obtained from Nanostructured & Amorphous Materials Inc. (Houston, TX, USA). All aqueous solutions were prepared using ultra-pure water (18.2 MΩ/cm) from a Milli-Q system (Merck Millipore, Burlington, MA, USA).

### 2.2. Preparation of TiO_2_/ZnO Heterojunctions

TiO_2_/ZnO heterojunction powdered catalysts were prepared using a simple physical dry-blending approach, as previously reported [25]. TiO_2_ and ZnO nanopowders were mechanically mixed at predetermined weight ratios of 7:3, 5:5, and 3:7, with pure TiO_2_ and ZnO formulations used as references. Therefore, the samples are denominated according to their relative ratios of each catalyst: 7T-3Z, 5T-5Z, and 3T-7Z for heterojunctions with weight ratios of TiO_2_ and ZnO of 7:3, 5:5, and 3:7, respectively. This processing method was selected to avoid the structural changes that can happen with more complex chemical synthesis, ensuring TiO_2_ and ZnO kept their properties [25].

### 2.3. Composite Membrane Preparation

The prepared powdered heterojunctions were used to fabricate PVDF-HFP and PVDF-TrFE composite membranes via TIPS and ES. In all formulations, the catalysts were incorporated at a fixed loading of 10 wt. % relative to the polymer to optimize performance without losing mechanical properties. For that, the required amount of TiO_2_, ZnO, or TiO_2_/ZnO was first dispersed in DMF under an ultrasonic bath for 3 h to ensure deagglomeration and full dispersion of the catalyst. Afterwards, the polymer was added to achieve a final concentration of 15 *v*/*v* %, and the mixture was stirred at 100 rpm until a complete homogeneous solution was achieved. For TIPS membranes, the solution was spread into a previously cleaned glass using a doctor-blade (850 μm thickness). Membranes were formed by solvent evaporation at ambient temperature over 24 h, adapting the TIPS method reported previously [26]. For ES membranes, the solution was loaded into a 10 mL syringe comprising a needle with a diameter of 0.5 mm. After an optimization process, electrospinning was conducted using 14 kV of voltage and a feed rate of 0.4 mL/h, and the fibers were collected in a metallic collector placed at 20 cm of distance from the needle. For improved nomenclature clarity, the samples are denominated according to the copolymer type: PVDF-HFP-based membranes are denominated as HFP, while PVDF-TrFE-based membranes are denominated as TrFE.

### 2.4. Physical–Chemical Characterization of the Catalysts and Composite Membranes

The surface morphology and elemental distribution of the samples were examined by scanning electron microscopy (SEM) coupled with energy-dispersive X-ray spectroscopy (EDS), using a Hitachi S-4800N microscope (Hitachi High-Technologies Corporation, Tokyo, Japan) equipped with an Oxford Instruments EDS detector (Oxford Instruments, Abingdon, UK). The specific surface area of the samples was determined from nitrogen adsorption–desorption measurements performed at 77 K using a Micromeritics TriStar II 3020 analyzer (Micromeritics Instrument Corporation, Norcross, GA, USA). The crystalline structure of the catalysts and composite membranes was analyzed by X-ray diffraction (XRD) using a Philips X’Pert PRO diffractometer (Philips Electronic Instruments, Almelo, The Netherlands) operating with Cu-Kα radiation (λ = 1.5418 Å) at 40 kV and 40 mA. Diffraction patterns were recorded in a θ–θ configuration over a 2θ range from 10° to 80°, using a step size of 0.026°. Chemical bonding and vibrational characteristics were studied by Fourier-transform infrared spectroscopy (FTIR) (Thermo Fisher Scientific, Waltham, MA, USA) in the range of 4000–600 cm^−1^. From the data, the relative fraction of the polymer β-phase in the membranes was calculated with Equation (1) [27]:

(1) $$F\left(\beta \right)=\frac{A_{\beta }}{\left(\frac{K_{\beta }}{K_{\alpha }}\right)A_{\alpha }+A_{\beta }}$$

where *A_α_* and *A_β_* are the absorbance of the characteristic absorption bands of the *α-* and *β*-phase of the polymer at 763 and 838 cm^−1^, respectively. *K_α_* and *K_β_* are the absorption coefficients for the given values (6.1 × 10^4^ and 7.7 × 10^4^ cm^2^/mol). The optical properties of the membranes were evaluated using UV–Vis-NIR diffuse reflectance spectroscopy (DRS), recorded in the wavelength range of 200–2000 nm with a Jasco analytical spectrophotometer (JASCO International Co., Ltd., Tokyo, Japan). The optical band gap energy (E_g_) was estimated from the obtained data using Kubelka–Munk (Equation (2)) and Tauc (Equation (3)) equations [12]:

(2) $$F\left(R\right)=\frac{{\left(1-R_{\infty }\right)}^{2}}{2R_{\infty }}$$

(3) [F(R)hv]1n=A(hv−Eg)

where *R_∞_* represents the reflectance of the sample relative to a non-absorbing reference (*R_sample_/R_reference_*). The bandgap was determined using the equation [*F(R)·hν*]^1^*^/n^* versus photon energy (*hν*), where *A* is a constant, *h* is the Planck’s constant (6.62 × 10^−34^ J/s), *ν* is the frequency, and *n* is a parameter that equals 2 for indirect semiconductors. The wettability of the membranes was determined using an Ossila L20041-UK [MR6.1] (Ossila Ltd., Sheffield, UK). Water was used as the probe liquid. Measurements were repeated at three different spots on the same membrane, and the mean value and the error were calculated.

### 2.5. Functional Evaluation of the Composite Membranes

The catalytic performance of the composite membranes was evaluated by degrading CIP under two conditions: *(i)* photocatalysis, and *(ii)* sono-enhanced photocatalysis. For the experiments, 250 mg (6 cm^2^ of area) of a composite membrane (with 25 mg, 10 wt. % of catalyst) was immersed in 50 mL of a CIP solution (5 mg/L, pH 4). The system was first stirred in the dark for 30 min to establish adsorption–desorption equilibrium. For photocatalytic tests, the beaker was then placed under a photoreactor (14 UV light lamps (6 W, 365 nm) and 3 visible-light lamps (60 W, 500 nm), total irradiance of 25 mW/cm^2^). For sono-enhanced photocatalytic tests, the beaker was placed in an ultrasonic bath (Elmasonic S 30, 37 kHz, 80 W, Elma Schmidbauer GmbH, Singen, Germany) with simultaneous light irradiation. In both setups, the solution was stirred in a 100 mL beaker with a fixed 13.5 cm distance from the light source. Aliquots were collected at defined time intervals (−30 to 360 min). The CIP concentration was monitored by UV–Vis spectrophotometry (Tecan Infinite^®^ 200 PRO, Tecan Group Ltd., Männedorf, Switzerland) and using a pre-established calibration curve. The synergistic effect was calculated by the relative enhancement factor (REF), according to Equation (3):

(4) $$REF=\frac{SPC}{PC}$$

where SPC and PC are the sono-enhanced photocatalytic and photocatalytic degradation efficiencies, respectively.

### 2.6. Antibacterial Activity of Composite Membranes

The antibacterial activity of the composite membranes was assessed using an agar diffusion (zone of inhibition) assay against four bacterial strains: *Staphylococcus aureus*, *Salmonella enterica*, *Listeria monocytogenes*, and *Escherichia coli*. Membrane fragments weighing between 1.5 and 5 mg were sterilized under UV light for 15 min and placed on nutrient agar plates previously inoculated with bacterial suspensions at a concentration of 10^5^ CFU/mL. The plates were incubated at 37 °C for 24 h, after which the diameters of the inhibition zones were measured and provided in millimeters.

## 3. Results and Discussion

### 3.1. Catalysts’ Characterization

The successful formation of a heterojunction relies fundamentally on the catalyst properties. The structural, morphological, and optical characteristics of the TiO_2_/ZnO heterojunction are crucial for the intended application, as they directly determine light absorption, piezoelectric activity, and charge separation efficiency. Therefore, pure ZnO and TiO_2_, and TiO_2_/ZnO composites were thoroughly characterized prior to their incorporation into the polymer matrix.

**Figure 1:**
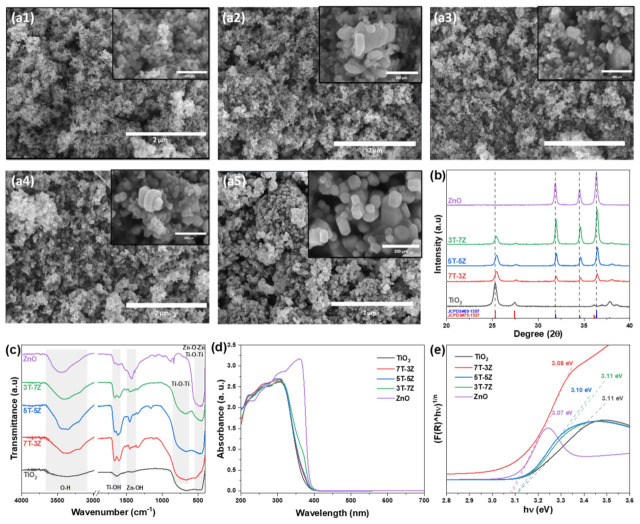
SEM images of (**a1**) TiO_2_, (**a2**) 7T-3Z, (**a3**) 5T-5Z, (**a4**) 3T-7Z, and (**a5**) ZnO; (**b**) XRD patterns; (**c**) FTIR spectra; (**d**) UV–Vis DRS spectra; and (**e**) transformed Kubelka–Munk vs. hν plot for all catalysts.

SEM analysis revealed distinct morphological differences between ZnO, TiO_2_ and TiO_2_/ZnO heterojunctions, as presented in Figure 1(a1–a5). ZnO nanoparticles exhibited a uniform, quasi-spherical morphology with moderate agglomeration, consistent with the isotropic growth habits of hexagonal wurtzite ZnO [28]. This well-defined structure provides an accessible surface area, a key factor for high photocatalytic efficiency. In contrast, the heterojunction samples showed more complex and densely packed structures. Similarly, TiO_2_ particles are thickly stacked, forming clusters due to the nanoscale size and high surface energy [29]. The ZnO-rich composite (3T-7Z) displayed a unique particle-within-particle morphology, suggesting intimate interfacial contact (inset of Figure 1(a4)). Although not quantified, this enhanced interface results in an increased agglomeration of nanoparticles, which usually reduces the effective surface area and accessibility of active sites compared to pure ZnO.

The crystal structures of the TiO_2_, ZnO, and TiO_2_/ZnO heterojunctions were elucidated by XRD (Figure 1b and Figure S1). The analysis confirms the successful formation of phase-pure and composite materials. The XRD pattern of TiO_2_ shows diffraction peaks at 25.3° (101), 37.8° (004), 48.1° (200), 53.9° (105), 55.1° (211), and 62.7° (204), characteristic of the anatase phase. In addition, smaller peaks at 27.4° (110) and 36.1° (101) were assigned to the rutile phase, in agreement with JCPDS #75-1537 [22]. For ZnO, sharp and intense diffraction peaks are observed at 2θ = 31.8° (100), 34.4° (002), 36.3° (101), 47.5° (102), 56.6° (110), 62.9° (103), 66.4° (200), and 69.1° (201), which are indexed to the hexagonal wurtzite structure (JCPDS #89-1397) [30]. The narrow full width at half maximum of these peaks demonstrates a high degree of crystallinity and minimal lattice strain within the ZnO nanocrystals. The formation of TiO_2_/ZnO heterojunctions is clearly evidenced by the evolution of the diffraction patterns. While the characteristic peaks of wurtzite ZnO remain present in all heterojunctions, they undergo a progressive reduction in intensity and noticeable broadening as the TiO_2_ content increases. Also, anodic shifts in the XRD peaks in the TiO_2_/ZnO heterojunctions are noted, where the most characteristic peak of TiO_2_ moves from 25.3º to 25.5º and ZnO reflections shift from 31.8, 34.5, and 36.2º to 32.0, 34.7, and 36.4º, confirming a lattice contraction within both phases. This structural change is driven by localized strains at the interface, originating from lattice interaction and ion diffusion during heterojunction formation [31]. Concurrently, the emergence of new, broad peaks at 25.3° and 48.0°, corresponding to the (101) and (200) planes of anatase TiO_2_, confirms its nanocrystalline nature. This conversion occurs because the high-energy mechanical processing introduces localized lattice defects and allows for trace diffusion of Zn^2+^ ions into the TiO_2_ surface [30]. The results confirm the coexistence of two distinct crystalline phases, which is essential for forming a heterojunction at their interface. This biphasic structure plays a crucial role in promoting charge separation and reducing e^−^/h^+^ recombination in photocatalytic processes [31].

The FTIR spectrum of the pristine ZnO powder (Figure 1c) shows a characteristic, intense absorption band centered at 500 cm^−1^, which is assigned to the stretching vibration of Zn–O bonds; also, an absorption band is noted in the range of 1400–1500 cm^−1^, ascribed to Zn–OH vibrations [32]. The spectrum of TiO_2_ exhibits a broad, intense absorption band in the range of 500–900 cm^−1^, attributed to the stretching vibration of Ti–O–Ti bonds in its crystal lattice; in addition, an absorption band is observed between 1600 and 1700 cm^−1^, related to Ti–OH vibrations [29]. The formation of TiO_2_/ZnO heterojunctions represents a combination of these previously mentioned bands: *(i)* the characteristic metal–oxygen vibration band of the pure oxides merge into a double, broad absorption envelope spanning approximately between 450 and 800 cm^−1^; *(ii)* the shape and profile of these broad bands are directly related to the relative amount of each isolated catalyst, and are a superposition of the pure oxide spectra; *(iii)* the metal–hydroxyl band follows the same tendency as the metal–oxygen vibration band, and its intensity is also related to the relative amount of each isolated catalyst. This indicator of heteronuclear Zn–O–Ti bond vibrations serves as a spectroscopic signature of chemical interaction, confirming lattice disturbance and the establishment of a coupled heterostructure [30].

The optical properties of TiO_2_, ZnO, and TiO_2_/ZnO heterojunctions were determined using UV–Vis DRS (Figure 1d). Pristine TiO_2_ reflects more than 90% of all of the radiation in the visible range (380–750 nm) while absorbing all of the radiation in the UV range (<380 nm), demonstrating its well-reported UV photoactivity. Pristine ZnO has a broader absorption spectrum, extending its light absorption up to 400 nm, entering the blue region of the visible-light spectrum. In addition, the absorption intensity increases when compared to the pristine TiO_2_. The heterojunctions have a similar profile to the one of TiO_2_. However, for the ZnO-rich heterojunction (3T-7Z), a significant fraction of light absorption is observed up to 400 nm, mimicking the behavior of pristine ZnO. From this data, the bandgap and apparent optical bandgap energies of the catalysts were determined (Figure 1e). The ZnO catalyst exhibits a bandgap of 3.07 eV, slightly lower than the typical bulk ZnO value of ~3.2 eV [30]. Similarly, the bandgap of TiO_2_ is 3.11 eV, which agrees with previous reports on this photocatalyst [22]. As for the heterojunctions, the introduction of TiO_2_ did not significantly change their optical properties. The 7T-3Z, 5T-5Z, and 3T-7Z heterojunctions showed a slight increase in the apparent optical bandgap energy compared to pristine ZnO from 3.07 to 3.08, 3.10, and 3.11 eV, respectively. This blue shift is consistent with the XRD-observed reduction in ZnO crystallite size, suggesting a quantum confinement effect as the dominant mechanism, rather than lattice substitution [33]. Despite similar bandgap and apparent optical bandgap energies, it is expected to have enhanced interfacial interactions promoting efficient charge transfer across the heterojunction interface. This enhanced visible-light absorption is hypothesized to originate from the creation of localized sub-bandgap or mid-gap states at the heterojunction boundaries. The extended visible-light absorption tail observed by UV–Vis DRS, combined with the structural lattice strain confirmed by XRD, strongly points toward such interfacial electronic modifications, which are widely documented in literature as a common effect in heterojunctions with strong interfacial contact and type II band alignment [31].

### 3.2. Composite Membranes’ Characterization

**Figure 2:**
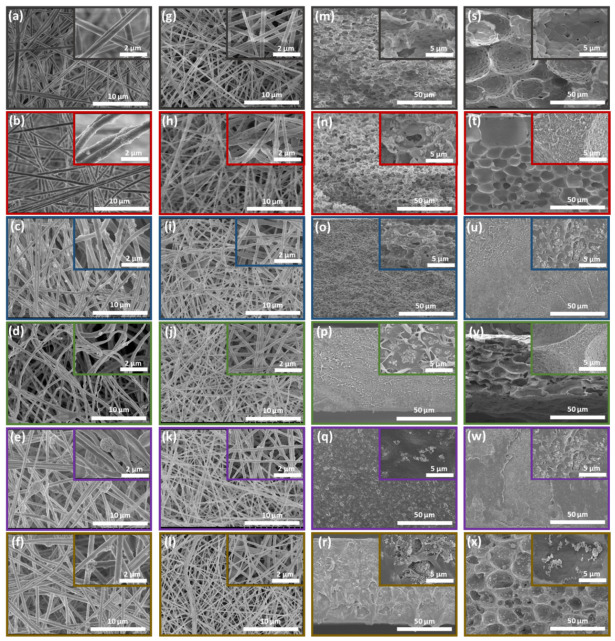
SEM images of (**a**–**l**) ES membranes: (**a**) HFP; (**b**) TiO_2_@HFP; (**c**) 7T-3Z@HFP; (**d**) 5T-5Z@HFP; (**e**) 3T-7Z@HFP; (**f**) ZnO@HFP; (**g**) TrFE; (**h**) TiO_2_@TrFE; (**i**) 7T-3Z@TrFE; (**j**) 5T-5Z@TrFE; (**k**) 3T-7Z@TrFE; (**l**) ZnO@TrFE. (**m**–**x**). TIPS membranes: (**m**) HFP; (**n**) TiO_2_@HFP; (**o**) 7T-3Z@HFP; (**p**) 5T-5Z@HFP; (**q**) 3T-7Z@HFP; (**r**) ZnO@HFP; (**s**) TrFE; (**t**) TiO_2_@TrFE; (**u**) 7T-3Z@TrFE; (**v**) 5T-5Z@TrFE; (**w**) 3T-7Z@TrFE; (**x**) ZnO@TrFE.

Following the synthesis of TiO_2_/ZnO heterojunctions, the next critical step was to engineer the polymer matrix. Moving beyond its traditional role as a passive support, the piezoelectric polymer was selected to be an active component in the hybrid system. Its morphology, crystalline phase, and surface properties are critical as they directly influence catalyst dispersion, light penetration, availability of active sites, and the piezoelectric response. SEM analysis was used to characterize the morphology of ES and TIPS membranes prepared with PVDF-HFP and PVDF-TrFE in their pristine form and with incorporated TiO_2_, ZnO, and TiO_2_/ZnO heterojunctions (Figure 2). The distinct morphologies of the membranes produced by the two routes are evidenced.

Pristine HFP and TrFE ES membranes form bead-free, non-woven networks with high porosity (80–90%) and strong interconnectivity [22]. HFP fibers are thicker than their TrFE counterparts (0.54 ± 0.08 µm vs. 0.33 ± 0.08 µm), which may be ascribed to the higher viscosity of the PVDF-HFP solution and lower dielectric constant [34]. Upon catalyst introduction, the uniform morphology was kept, though specific heterojunctions on HFP samples (5T-5Z and 3T-7Z) induced bead-on-string structures and fiber curvature, indicating electrospinning jet instability [21]. In contrast, the TrFE ES membranes consistently preserved smooth, continuous structures with nanoscale porosity regardless of the catalyst, a characteristic attributed to its higher crystallinity and distinct viscoelastic properties [35].

For the TIPS membranes, pristine HFP membrane exhibits a sponge-like, macroporous morphology with a highly porous structure (70–75%) and pore size of 4 ± 2 µm, characteristic of TIPS-derived matrixes, where liquid–liquid phase separation during solvent evaporation produces an interconnected porous polymer matrix [21]. The pristine TrFE membrane presents a smoother porous surface with an even higher porous structure (75–80%) and pore size (10 ± 2 µm), reflecting intrinsic differences in crystallinity and phase-separation behavior between the two co-polymers [36]. Integration of TiO_2_/ZnO heterojunctions increased the porosity and pore size of the membranes, and a more defined interconnected pore structure is observed. This morphological difference is attributed to the distinct physicochemical roles of catalysts and polymers during membrane processing. ZnO is known to promote β-phase crystallization in PVDF matrixes, while surface hydroxyl groups of TiO_2_ can improve filler dispersion and solvent–filler interactions, and influence phase separation dynamics [37].

**Figure 3:**
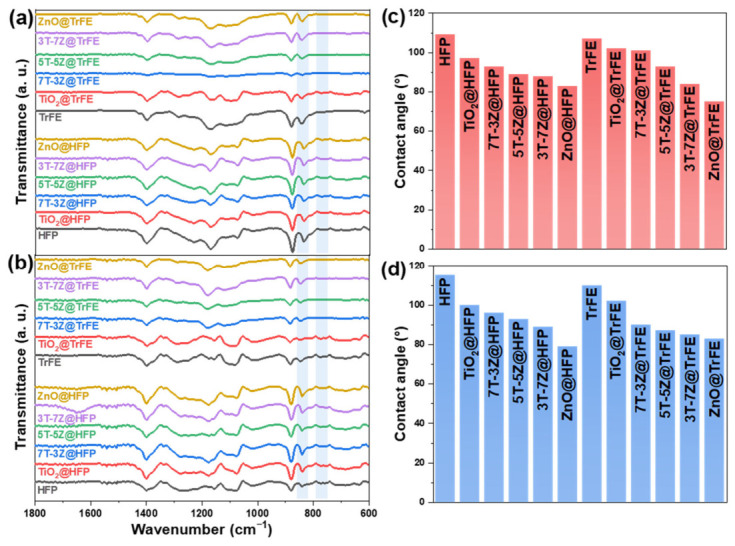
FTIR spectra of (**a**) TIPS and (**b**) ES membranes; contact angle values of (**c**) TIPS and (**d**) ES membranes.

To confirm the successful incorporation of the catalysts and their impact on the polymer matrix, FTIR analysis was performed (Figure 3a,b). All spectra disclose the characteristic absorption bands of PVDF: in the 1400–1000 cm^−1^ region, ascribed to C–F stretching vibrations, with specific peak features allowing for differentiation between HFP and TrFE copolymers; and bands at 840 and 760 cm^−1^ related to the β and α phases of PVDF, respectively [21]. After catalyst incorporation, the presence of a characteristic metal–oxygen bond (M–O) vibration band in the 400–700 cm^−1^ region for all composite membranes, and absent in the pristine membranes, represents a direct evidence that the catalysts are embedded within the polymer matrix [38]. Furthermore, a pronounced and broad O–H stretching band at ~3300 cm^−1^ was observed for all composite membranes, indicating the presence of surface hydroxyl groups [22]. The β-phase fraction, which is responsible for the piezoelectric response of PVDF-based materials, was calculated from the FTIR data (Table 1). The calculated β-phase fractions confirm a pronounced catalyst-induced crystallization effect. The incorporation of TiO_2_, ZnO, and TiO_2_/ZnO heterojunctions significantly favored the electroactive β-phase in both polymers and processes. For HFP, the β-phase fraction increased from 52% in pristine form to over 80% in composite membranes. A more pronounced enhancement was observed for TrFE, where catalyst incorporation raised the β-phase to 98% for the 7T-3Z@TrFE TIPS membrane. This improvement is attributed to strong interfacial interactions between the polar surfaces of the metal oxides and the CF_2_ dipoles of the polymer chains, which stabilize β-phase chain conformation [39].

The wettability of the composite membranes was assessed via contact angle measurements (Figure 3c,d). The pristine HFP and TrFE ES membranes exhibited high contact angles of 116° and 109°, respectively, confirming intrinsic hydrophobicity typical of PVDF. Incorporating catalysts noticeably changes this property, reducing hydrophobicity in a composition-dependent manner. HFP ES membranes showed a systematic reduction to 77°, while TrFE ES membranes decreased to 85° with ZnO-rich heterojunctions. A similar tendency was observed for the TIPS membranes, regardless of polymer type. This decrease, consistent with the FTIR evidence of enhanced O-H presence, reflects a pronounced increase in surface hydrophilicity, crucial for facilitating aqueous contaminant access to catalytic sites. In sum, catalyst incorporation achieved a dual optimization: it significantly enhanced the piezoelectric β-phase content, readying the composite membrane for ultrasonic activation, while simultaneously creating a more hydrophilic surface to improve interfacial contact with the aqueous contaminant solution.

### 3.3. Functional Evaluation of the Composite Membranes

The physical and chemical characterization of a membrane provides a necessary foundation, yet it remains an insufficient predictor of its efficiency in water remediation. A persistent challenge in catalysis is the potential for catalytic particles to become inaccessible within the polymer matrix. Consequently, direct performance evaluation is crucial.

All catalytic experiments were carried out at pH 4. CIP speciation dictates its charge: cationic below pK_a1_ (6.1), zwitterionic between 6.1 and 8.9, and anionic above pK_a2_ (8.9) [40]. Electrostatic attraction between the CIP species and the catalyst is pH-dependent, and the surface charge directly governs adsorption capability, significantly accelerating or suppressing subsequent ROS-mediated degradation kinetics and overall remediation efficiency [41].

Firstly, control experiments were carried out to understand the photolysis and adsorption role in the overall performance of the composite membranes. CIP undergoes negligible photolysis, with less than 5% degradation observed under both UV and visible-light irradiation in the absence of a catalyst. With respect to adsorption, pristine HFP and TrFE membranes exhibited minimal affinity for CIP, with adsorption capacities below 7%. Incorporating the catalysts, particularly the TiO_2_/ZnO heterojunctions, led to a measurable increase in adsorption, with efficiencies ranging from 3 to 19%. This enhancement is attributed to the introduction of polar surface sites and increased surface area provided by the dispersed catalysts.

#### 3.3.1. Photocatalytic CIP Degradation

**Figure 4:**
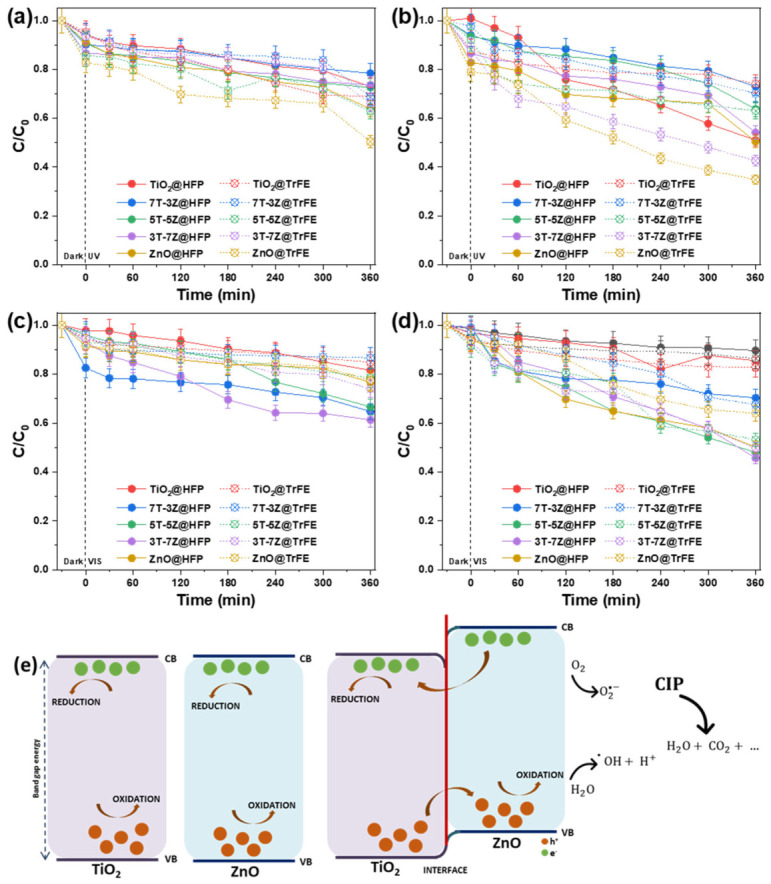
Photocatalytic CIP degradation (**a**,**b**) under UV irradiation by: (**a**) TIPS and (**b**) ES membranes; (**c**,**d**) under visible-light irradiation by: (**c**) TIPS and (**d**) ES membranes ([CIP] = 5 mg/L; pH = 4; time: 360 min). (**e**) Schematic band diagram and mechanism of enhanced performance of TiO_2_, ZnO, and TiO_2_/ZnO heterojunctions.

The photocatalytic activity of the prepared composite membranes was evaluated under UV (Figure 4a,b) and visible-light irradiation (Figure 4c,d). Control experiments using pristine polymeric membranes show a slight CIP removal (<19%), confirming that photoactive catalysts are key to drive the degradation process.

Firstly, the TIPS-produced membranes were compared by evaluating the effect of both the polymeric matrix (HFP vs. TrFE) and the catalyst type (TiO_2_ vs. ZnO vs. TiO_2_/ZnO heterojunctions) under UV irradiation (Figure 4a). When TiO_2_ was incorporated into the membrane, the degradation efficiencies increased to 27 and 31% for the HFP and TrFE membranes, respectively. On the other hand, in composite membranes containing ZnO, degradation efficiencies of 36 and 50% were achieved for HFP and TrFE membranes, respectively. When TiO_2_/ZnO heterojunctions were incorporated, efficiencies ranging from 22 to 27% were obtained. With respect to the ES processing method (Figure 4b), TiO_2_-based membranes showed efficiencies of 49 and 26% for HFP and TrFE membranes, respectively; ZnO-based membranes achieved 52 and 65% for HFP and TrFE membranes, respectively; and TiO_2_/ZnO-based membranes presented efficiencies ranging from 27 to 57%. Globally, two main conclusions can be withdrawn: Firstly, TrFE composite membranes and the ES method consistently demonstrated higher degradation efficiency than their HFP counterparts and the TIPS method, being the best-performance membrane the ZnO@TrFE composite membrane prepared by ES (65%). This enhanced performance is attributed to two different phenomena: *(i)* the higher porosity of PVDF-TrFE composite membranes allows for a faster and more efficient interaction of water and CIP molecules with the catalyst present on the pores’ surface, favoring adsorption in the first stage and the later reaction with the photogenerated ROS [22]; *(ii)* ES composite membranes show a higher surface area and interconnected porous network, which improve catalyst accessibility and photon absorption [42]. Secondly, regardless of the polymer matrix and the processing method, composite membranes containing ZnO as the catalyst always outperform the remaining catalysts (65% vs. 26, 30, 37, and 57% for ZnO, TiO_2_, 7T-3Z, 5T-5Z, and 3T-7Z@TrFE ES, respectively). Although both TiO_2_ and ZnO are standard UV photocatalysts, the superior efficiency of ZnO for CIP degradation under UV irradiation may be related to its higher electronic mobility and faster charge transport, minimizing e^-^/h^+^ recombination [43]. It is important to note that, under UV radiation, all catalysts have optimal optical properties for catalytic activity.

Under visible-light irradiation (Figure 4c,d), the photocatalytic behavior presents different tendencies. For TIPS membranes (Figure 4c), HFP-based composite membranes exhibit enhanced performance compared to their TrFE counterparts (e.g., 39 vs. 26% for 7T-3Z-based HFP and TrFE membranes, respectively). A similar tendency is observed for ES membranes (Figure 4d), while not so evident as in the previous case (e.g., 54 vs. 50% for 7T-3Z-based HFP and TrFE membranes, respectively). As described previously, TrFE-based membranes are characterized by higher porosity compared to their HFP counterparts, which should yield higher efficiencies for these membranes. Our hypothesis is that due to the lower energy associated with visible radiation, the driven force in these experiments is the catalyst itself rather than the catalyst–polymer interaction and compatibility. Actually, the key difference when comparing the experiments performed under UV and VIS irradiation is the degradation efficiency shown by the different catalysts. Under VIS irradiation, the single catalysts are not able to degrade CIP to a similar extent compared to that under UV radiation. Under UV, pristine ZnO and TiO_2_ have similar or even higher performances when compared to the heterojunctions, while under VIS irradiation, their efficiency drops significantly (e.g., from 49 to 15% for TiO_2_@HFP ES membranes, under UV and VIS, respectively). Here, the heterojunctions stand out as the most efficient catalyst for visible-driven photocatalysis. The optimal TiO_2_/ZnO ratio is polymer-dependent, and across all polymer matrixes and processing methods, the ZnO-rich heterojunction (3T-7Z) achieved higher performances (39, 26, 54, and 50% for HFP TIPS, TrFE TIPS, TrFE ES, and TrFE ES, respectively). This is attributed to their high bandgap (3.11 and 3.07 eV for TiO_2_ and ZnO, respectively), which hinders light absorption in the visible region of the spectrum. In addition, the higher β-phase content and the lower hydrophobicity of ZnO-rich composite membranes promote a better contaminant–catalyst interaction, increasing efficiency.

The degradation rate is related to the availability of the photocatalyst surface to generate e^−^/h^+^ pairs, which produces ROS. The CIP photocatalytic degradation rate fits a pseudo-first-order reaction (Figure S2). Like degradation efficiency, the presence of the catalyst leads to a higher degradation rate (Tables S1 and S2). TIPS and ES membranes without a catalyst present a negligible CIP degradation rate, both under UV and VIS (<10^−5^ min^−1^). The incorporation of catalyst results in a 10-to-100-fold increase in the degradation rate. All composite membranes present degradation rates higher than 1 *×* 10^−4^ min^−1^, being the highest degradation rates under UV and VIS obtained for 3T-7Z@TrFE composite membranes (0.0026 and 0.0019 min^−1^, respectively). When comparing composite membranes, TrFE-based membranes outperform their HFP counterparts under UV irradiation, but the opposite happens under visible-light irradiation. Common to both light sources, ES-based membranes always outperform their TIPS-based counterparts.

Based on the catalytic enhancements and alignment constraints, the TiO_2_/ZnO system is proposed to form a staggered type II heterojunction configuration [29] (Figure 4e). According to established literature values, pristine anatase TiO_2_ typically presents a conduction band (CB) edge around −0.2 to −0.3 V and a valence band (VB) edge around 2.9 to 3.0 V, while wurtzite ZnO has a slightly higher CB around −0.3 to −0.4 V and a VB edge near 2.8 V (vs. NHE). This energetic balance creates a favorable thermodynamic gradient for charge migration. Under irradiation, photo-generated electrons are assumed to migrate from the more negative CB of ZnO to the CB of TiO_2_, while holes accumulate in the higher VB of ZnO [14]. This separation is expected to suppress charge carrier recombination, extending carrier lifetimes for surface redox reactions [44]. Although both components are wide-bandgap semiconductors naturally responsive to UV light, the close contact between the two phases (as seen previously by XRD) is proposed to create sub-bandgap interfacial defect states. This localized energy state creation shifts the absorption threshold, enabling enhanced visible-light absorption and driving ROS generation.

Overall, these results establish that photocatalytic degradation in these composite membranes arises from an interplay between polymeric matrix, membrane morphology, and catalyst design. This baseline provides a reference for evaluating ultrasonic synergy. Among all tested composite membranes, the 3T-7Z@HFP ES membrane emerges as the most promising candidate for sono-enhanced photocatalysis.

#### 3.3.2. Sono-Enhanced Photocatalytic CIP Degradation

**Figure 5:**
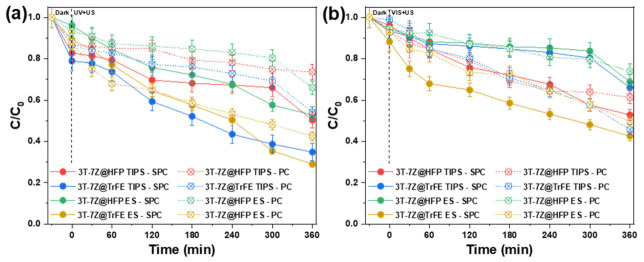
Sono-enhanced photocatalytic CIP degradation under (**a**) UV and (**b**) VIS irradiation by the top-performance 3T-7Z@-based membranes ([CIP] = 5 mg/L; pH = 4; time: 360 min).

To evaluate potential synergistic effects, sono-enhanced photocatalytic CIP degradation was investigated by combining ultrasonic vibrations and light irradiation using all composite membranes containing 3T-7Z as the catalyst (Figure 5). Building upon the established photocatalytic baseline, this study aimed to determine whether sonocatalytic activation could enhance degradation efficiency through improved charge separation and mass transfer.

Control experiments with pristine HFP and TrFE membranes, both under UV and VIS irradiation, confirmed negligible CIP degradation (<5%), highlighting the essential role of the catalyst. Under ultrasonic stimulation and UV irradiation (Figure 5a), the effect of the polymer matrix is evident. The TrFE-based composite membranes outperform their HFP counterparts using both processing methods. Here, the highest performance of 71% was achieved for the 3T-7Z@TrFE ES composite membrane. A similar clear tendency is shown when comparing the processing method, as besides the clear increased efficiency of TrFE-based membranes, the superior efficiency of ES membranes when compared to TIPS membranes is also evident (47 and 71%, respectively, for 3T-7Z@TrFE). Under ultrasound stimulation and visible-light irradiation, similar trends were observed. The TrFE-based membranes show superior efficiency compared to the HFP counterparts in all cases, regardless of the processing method. In this case, the highest efficiency was achieved for the 3T-7Z@TrFE composite membrane prepared by ES. Regarding the processing method, a similar observation is seen. ES composite membranes present higher efficiency than TIPS composite membranes, both when using HFP and TrFE as polymer matrixes. These two main results are ascribed to two key physical–chemical aspects: *(i)* the superior performance of TrFE is explained by its higher crystalline *β*-phase content and piezoelectric coefficient compared to HFP (25–40 vs. 15–25 pC/N for TrFE and HFP, respectively [21]), which can generate stronger surface potentials under mechanical stress, boosting ROS generation [45]; *(ii)* the superior efficiency of ES membranes, as explained previously, is related to their higher surface area and interconnected porous network, which enhances the number of active sites for CIP degradation.

Apart from direct comparisons between polymer matrix and processing method, increased efficiency of the sono-enhanced photocatalytic process when compared with using the single photocatalysis was observed (solid vs. dash lines in Figure 5). The relative enhancement factor (REF) values, calculated from Equation (3), confirm the presence of synergistic effects in the sono-enhanced photocatalytic process for all cases (Table 2).

As noted previously with photocatalytic CIP degradation, under sono-enhanced photocatalysis, the presence of the catalyst also leads to a higher degradation rate (Tables S3 and S4). All composite membranes present degradation rates higher than 7 *×* 10^−4^ min^−1^, a 7-fold increase when compared with the single photocatalytic method. The highest degradation rate under UV was obtained for 3T-7Z@TrFE ES composite membrane (0.0032 min^−1^), representing a 5-fold increase compared to the single photocatalytic method. Under visible-light irradiation, the degradation rates are always higher when compared to the photocatalytic method, except for the 3T-7Z@HFP ES composite membrane. Our hypothesis is that cavitation bubbles during sono-enhanced photocatalysis may physically damage the polymer matrix, scatter incoming light, or cleave the TiO_2_/ZnO active sites. This would destroy the heterojunction network and limit light absorption, resulting in lower performance compared to photocatalysis.

From the data, there is an interesting aspect to highlight: the REF values reached their maximum with the TIPS-based composite membranes, despite their lower overall degradation efficiency. The highest REF values (1.38 and 1.31) were obtained for the 3T-7Z@TrFE and 3T-7Z@HFP membranes, respectively, demonstrating the significant contribution of ultrasound-driven piezoelectric activation. The PVDF matrix effectively transmits ultrasonic deformation (cavitational stress) to the immobilized heterojunction, boosting their internal piezoelectric potential (on the ZnO side), which, in turn, enhances charge separation and ROS generation13. These higher REF values can also be explained by the significantly lower performance of TIPS membranes under photocatalysis, as their lower porosity and surface area did not allow for an efficient CIP–catalyst interaction. To some extent, these constrains are overcome with the presence of ultrasound, as the piezoelectricity associated with the polymer enhances ROS generation, boosting sono-enhanced photocatalytic activity [19]. For TrFE-based composite membranes, the lower REF values are explained by their higher efficiency by photocatalysis because of the higher surface area and porosity, as discussed previously.

Overall, the significant REF values strongly support a synergistic interplay between photocatalytic and piezocatalytic pathways for the composite membrane under UV and visible light. This highlights the potential of coupling strongly piezoelectric semiconductors with a flexible polymer matrix to maximize performance in hybrid sono-enhanced photocatalytic systems. This study demonstrates that optimal sono-enhanced photocatalytic performance is achieved by coupling three key factors: *(i)* a flexible polymer matrix (PVDF-based co-polymers) for effective strain transduction; *(ii)* an effective heterojunction catalyst tailored for coupling visible-light absorption and piezoelectric activation; and *(iii)* a high surface-area fibrous architecture (ES membranes) for improved accessibility and mass transfer. By coupling all of this, the 3T-7Z@TrFE ES composite membrane delivered the highest degradation efficiencies both under UV and visible-light irradiation (71 and 57%, respectively).

Similar conclusions can be drawn when comparing the REF values obtained from the degradation rates (Table S5). Significant REF values support our previous claim that there is a synergy between photoactive and piezoelectric materials under UV and visible light. Apart from the 3T-7Z@HFP ES composite membrane under visible-light irradiation, all composite membranes present a significant synergy with maximum REF values of 2.36 and 1.25 under UV and visible, respectively, when employing the 3T-7Z@HFP ES and 3T-7Z@TrFE ES composite membranes, respectively. These findings establish these material combinations as robust and efficient platforms for advanced sono-enhanced photocatalytic water purification across different irradiation conditions.

### 3.4. Insights on the Degradation Mechanism

This study compared two fabrication strategies for producing PVDF-based composite membranes aimed at CIP degradation via photocatalytic and sono-enhanced photocatalytic processes. The results revealed that ES membranes, particularly those composed of TrFE, exhibited superior performance. This enhancement arises from the fibers’ high porosity, interconnected network, and uniform distribution of catalyst nanoparticles, which increase catalytic sites and improve light penetration. These features significantly accelerated CIP degradation under both UV and visible light. The introduction of ultrasound during sono-enhanced photocatalysis further improved efficiency. Acoustic cavitation and mechanical vibrations promote the separation of photogenerated charges, especially in piezoelectric semiconductors such as ZnO [13]. This enhances ROS generation, including ^•^OH, O_2_^−•^, and hydroperoxyl radicals (HO_2_^•^), which attack the aromatic and heterocyclic rings of CIP [43].

**Figure 6:**
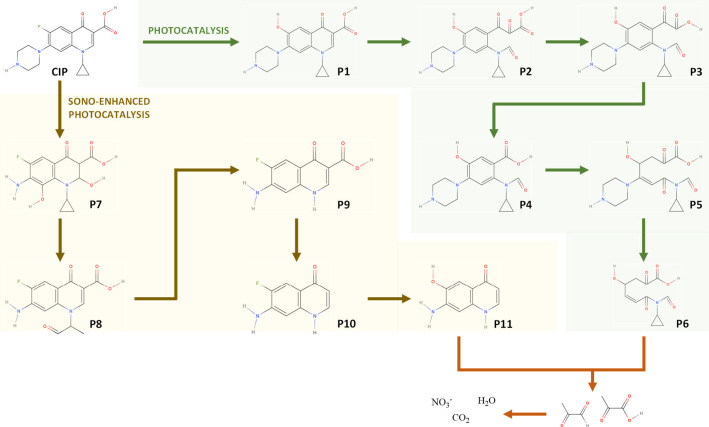
Proposed degradation mechanism for CIP using the 3T-7Z@TrFE composite membrane under photocatalysis and sono-enhanced photocatalysis.

A photocatalytic degradation mechanism for CIP was proposed, based on previous works using similar materials and processes. This proposed degradation mechanism follows a conventional semiconductor mechanism: photon absorption generates e^−^/h^+^ pairs, which react with water and oxygen to produce ROS capable of oxidizing CIP. Hydroxyl radicals play a dominant role, attacking the quinolone backbone and piperazine ring, progressively fragmenting intermediates into small organic acids before mineralization into CO_2_ and water [33]. Degradation pathways are proposed for both photocatalytic and sono-enhanced photocatalytic processes, as the energy provided by light and ultrasound affects the degradation processes by generating different types of ROS (Figure 6).

It is our hypothesis that photocatalytic CIP degradation follows a complex series of redox reactions, primary targeting the fluorine atom, the benzene ring, and the quinolone moiety [46,47,48]. In the standard photocatalytic pathway (green pathway), degradation starts with a defluorination step, transforming CIP into P1. Then, oxidative reactions in the quinolone moiety break the C=C bond near the carboxylic acid group, leading to P2. Subsequent steps involve the decarboxylation of the quinolone core and protonation, leading first to P3 and finally to P4. After that, a cleavage of the benzene ring happens to generate P5. Finally, the loss of the piperazine ring leads to P6. As the piperazine ring is stripped away, the remaining organic fragments are gradually oxidized into simple hydrocarbons before reaching complete mineralization into H_2_O, NO_3_^−^, and CO_2_ [12]. In contrast, sono-enhanced photocatalytic CIP degradation shifts the reactive focus (yellow pathway). This process focuses mostly on the hydroxylation and subsequent fragmentation of the piperazine ring. While this pathway also involves carboxylation and defluorination, the sequence of bond cleavage diverges significantly. The first degradation steps are the hydroxylation and cleavage of C–N and C–C bonds within the piperazine ring, generating P7. After that, P8 is generated though the carboxylation of the cyclopropane ring linked to the nitrogen atom in the quinolone moiety. This carboxylated by-product is subsequently decarboxylated to P9, and subsequent decarboxylation processes lead to P10. As the final step, the defluorination of the quinolone moiety leads to P11. The final redox reactions are like the photocatalytic pathway, with reactions leading to smaller acids, and ultimately complete mineralization into H_2_O, NO_3_^−^, and CO_2_ [49].

### 3.5. Antibacterial Activity

In addition to catalytic degradation, the real-world implementation of composite membranes in water remediation is reliant on their capacity to mitigate membrane fouling, which is a result of the deposition of organic matter on their surface, and particularly bacteria. The antibacterial performance of the prepared membranes was assessed using the agar diffusion assay against *S. aureus*, *S. enterica*, *L. monocytogenes*, and *E. coli* (Table 3).

As shown in Table 3, antibacterial activity under static conditions (no ultrasound or UV irradiation) was detected exclusively against *S. aureus*, whereas no inhibitory effect was observed for the other bacterial strains. Both TrFE and HFP membranes produced clear inhibition zones against *S. aureus*, measuring 14 mm and 16 mm, respectively.

The selective antibacterial activity of ZnO/TiO_2_@PVDF composite membranes is primarily related to the structural differences between bacterial cell walls. *S. aureus*, a Gram-positive bacterium, presents a thick but highly porous peptidoglycan layer without an outer protective membrane. This allows photo-generated ROS and released Zn^2+^ ions to penetrate and disrupt the cytoplasmic membrane [50]. Conversely, Gram-negative bacteria like *E. coli* and *S. enterica* have a complex outer membrane rich in lipopolysaccharides, which acts as a physical and chemical shield against oxidative stress [51]. Furthermore, these species often possess antioxidant enzymes (like catalase) that actively neutralize ROS and expel toxic metal ions. Although *L. monocytogenes* is Gram-positive, it is remarkably resilient due to specific stress-response genes that stabilize its membrane against lipid peroxidation [52]. Consequently, while the membrane successfully inactivates *S. aureus*, the ROS generation under ambient conditions by the heterojunction is often insufficient to overcome the advanced defense mechanisms of the other three pathogens.

## 4. Conclusions

This work demonstrated the engineering of multifunctional TiO_2_/ZnO heterojunction-loaded PVDF-based composite membranes for CIP degradation from water. By evaluating the interplay between catalyst composition (TiO_2_:ZnO ratios), polymer type (HFP vs. TrFE), and processing method (ES vs. TIPS), this work established the critical parameters to maximize both photocatalytic and sono-enhanced photocatalytic efficiencies.

Characterization of the catalysts confirmed the successful formation of type II TiO_2_/ZnO heterojunctions with a staggered band alignment, with the coexistence of typical anatase–rutile mixed phase of cubic TiO_2_ and hexagonal wurtzite ZnO. While structural and morphological properties were similar, this biphasic structure reduced the bandgap energy from 3.11 to 3.07 eV. Morphological analysis confirmed that ES produced architectures with higher porosity, more uniform catalyst dispersion, and interconnected network compared to TIPS. Structural characterization verified the retention of characteristic C–F vibrational modes and revealed the emergence of hydrophilic and oxidative groups after light exposure, both critical for improved wettability and catalytic activity. A critical finding was the catalyst-induced crystallization of the polymer: the incorporation of TiO_2_/ZnO heterojunctions acted as a nucleating agent, raising the piezoelectric β-phase fraction to 80% for HFP and 98% for TrFE. Furthermore, the presence of surface hydroxyl groups on the catalysts significantly shifted the membrane wettability from hydrophobic (116º) to more hydrophilic (77º), ensuring better access for water contaminants to the catalytic sites.

Photocatalytic experiments revealed that, while ZnO-based membranes show good performance under UV light—highest efficiency of 65% for ZnO@TrFE ES—the 3T-7Z heterojunction was the most effective for visible-light driven degradation (54% for 3T-7Z@TrFE ES). This was attributed to the sub-bandgap states that facilitate visible-light absorption and charge carrier mobility. The transition to sono-enhanced photocatalysis under UV irradiation introduced a powerful synergistic effect, enhancing the performance of the 3T-7Z@TrFE ES composite membrane from 65 to 74%. Under visible-light irradiation, an enhancement in efficiency was equally observed for all samples. With respect to REF, it reached values as high as 1.38, demonstrating that ultrasonic cavitation effectively triggers the piezoelectric potential of the PVDF matrix and boosts the internal piezoelectric potential of the ZnO side of the heterojunction, enhancing ROS generation. The degradation mechanism shifted notably under the hybrid method. While standard photocatalysis targets the quinolone moiety, the sono-enhanced photocatalytic pathway prioritizes the early fragmentation of the piperazine ring. This shift is vital for sustainability, as the piperazine derivatives are often the most toxic CIP intermediates. By degrading these harmful rings in the early stages, the hybrid process ensures a more environmentally friendly mineralization. In addition, the composite membranes displayed selective antibacterial activity. Strong inhibition zones were observed for *S. aureus* (16 and 14 mm for 3T-7Z@HFP and 3T-7Z@HFP, respectively), where the porous Gram-positive cell wall allowed ROS and Zn^2+^ ions to penetrate.

In summary, 3T-7Z@TrFE ES membranes are promising platforms for advanced water remediation. By coupling a high-surface-area fibrous architecture with a tailored type II heterojunction and a highly piezoelectric polymer matrix, this work developed a robust system capable of harnessing the full solar spectrum and ultrasonic energy. This research advances the field by merging piezoelectric effects with photocatalytic processes, providing a roadmap to develop new effective and sustainable systems for CEC degradation from water.

## Figures and Tables

**Table 1:** β-phase content of all prepared membranes.

	β-Phase Content (%)											
	HFP						TrFE					
	-	TiO_2_	7T-3Z	5T-5Z	3T-7Z	ZnO	-	TiO_2_	7T-3Z	5T-5Z	3T-7Z	ZnO
TIPS	53 ± 1	79 ± 2	77 ± 2	83 ± 2	83 ± 2	86 ± 2	61 ± 1	82 ± 2	98 ± 2	90 ± 2	75 ± 2	94 ± 2
ES	71 ± 1	74 ± 1	82 ± 2	75 ± 2	80 ± 2	81 ± 2	76 ± 2	58 ± 1	89 ± 2	95 ± 2	93 ± 2	94 ± 2

**Table 2:** Degradation efficiencies under PC and SPC, and calculated REF for the 3T-7Z-based composite membranes.

Polymer	Method	UV			VIS		
		Efficiency (%)		REF	Efficiency (%)		REF
		PC	SPC		PC	SPC	
PVDF-HFP	TIPS	26	34	1.31	39	47	1.21
	ES	46	57	1.24	54	34	0.63
PVDF-TrFE	TIPS	34	47	1.38	26	31	1.19
	ES	57	71	1.25	50	57	1.14

**Table 3:** Antibacterial activity (agar diffusion assay, zone of inhibition) of 3T-7Z ES composite membranes against four different bacteria.

Polymer	Inhibition Zone (mm)			
	*S. aureus*	*S. enterica*	*L. monocytogenes*	*E. coli*
PVDF-TrFE	14	-	-	-
PVDF-HFP	16	-	-	-

## Data Availability

The raw data supporting the conclusions of this article will be made available by the authors on request.

## Supplementary Materials

The following supporting information can be downloaded at: https://www.mdpi.com/article/10.3390/polym18131643/s1, Figure S1: XRD pattern of all catalysts; Figure S2: Degradation rate for the photocatalytic CIP degradation (a,b) under UV irradiation by: (a) TIPS and (b) ES membranes; (c,d) under visible light irradiation by: (c) TIPS and (d) ES membranes; Figure S3: Degradation rate for the photocatalytic CIP degradation (a,b) under UV irradiation and (c,d) under visible light irradiation by the top-performance 3T-7Z@-based membrane; Table S1: Reaction rate (kapp) for composite membranes by photocatalysis under UV irradiation; Table S2: Reaction rate (kapp) for composite membranes by photocatalysis under visible light irradiation; Table S3: Reaction rate (kapp) for composite membranes by sonophotocatalysis under UV irradiation; Table S4: Reaction rate (kapp) for composite membranes by sonophotocatalysis under visible light irradiation; Table S5: Degradation rates under PC and SPC, and calculated REF for the 3T-7Z-based composite membranes.
