# Supplementary material for: Synergistic Sono-Enhanced Photocatalytic Degradation of Antibiotics: Unlocking the Potential of Heterojunctions and Piezoactive Composite Membranes
Source: Polymers (Basel). 2026 Jul 1;18(13):1643. doi: 10.3390/polym18131643 (PMC13364294; doi:10.3390/polym18131643)
Supplement: Supplementary file 1 [file polymers-18-01643-s001.zip › polymers-4322443-supplementary.pdf]

Synergistic Sono-Enhanced Photocatalytic Degradation of Antibiotics: Unlocking the Potential of Heterojunctions and Piezoactive Composite Membranes

Samar Ben Atig[1,2,3] , Bruna F. Gonçalves[2], Moufida Chaari[4], Samia Dhahri[1,5], Hugo Salazar[2,*], Fathi Jomni[1], Senentxu Lanceros-Mendez[2,6,7]

[1] Laboratory of Materials Organization and Properties (LMOP), Faculty of Sciences of Tunis, University of Tunis El Manar, LR99ES17, Tunis, 2092, Tunisia
[2]BCMaterials, Basque Center for Materials, Applications and Nanostructures, UPV/EHU Science Park, 48940 Leioa, Spain
[3]Faculty of Science and Technology, University of the Basque Country, 48940 Leioa, Spain
[4]Laboratory of Microbial and Enzymatic Biotechnologies and Biomolecules (LBMEB), Center of Biotechnology of Sfax, University of Sfax, 3029, Tunisia
[5]Higher School of Health Sciences and Techniques of Tunis, University of Tunis El Manar, Tunisia
[6]IKERBASQUE, Basque Foundation for Science, 48009 Bilbao, Spain
[7]Physics Centre of Minho and Porto Universities (CF-UM-UP) and Laboratory of Physics for Materials and Emergent Technologies (LapMET), University of Minho 4710-057 Braga, Portugal

*Corresponding author: hugo.salazar@bcmaterials.net

Supporting Information

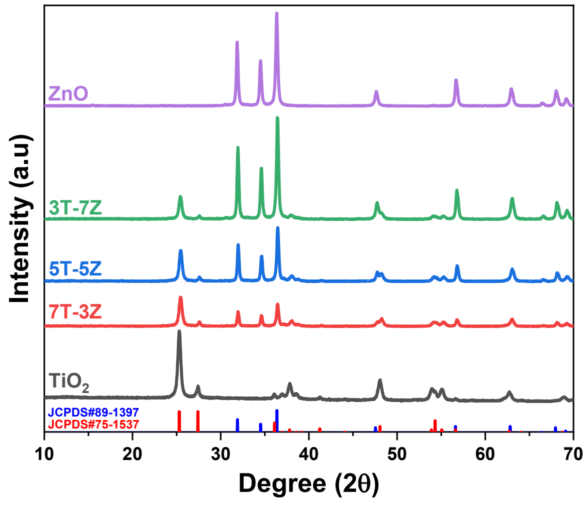

Figure S1. XRD pattern of all catalysts.

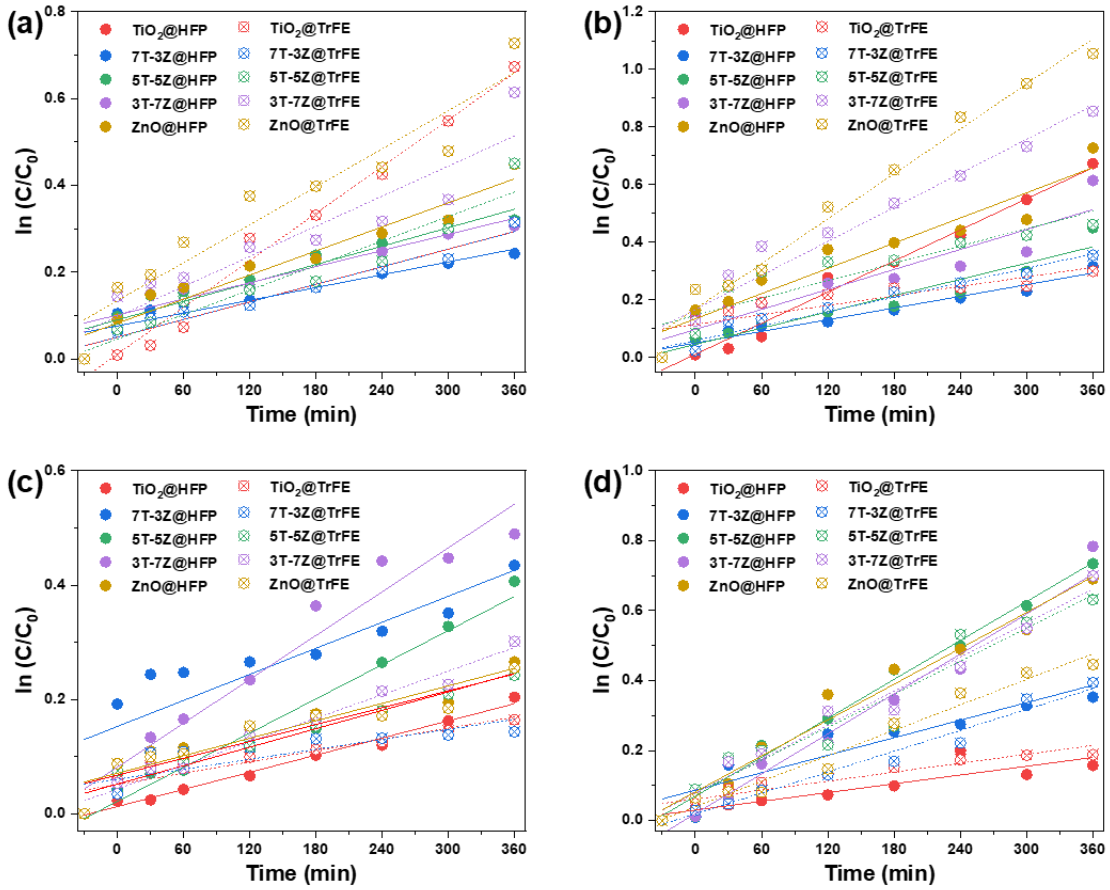

Figure S2. Degradation rate for the photocatalytic CIP degradation **(a–b)** under UV irradiation by: **(a)** TIPS and **(b)** ES membranes; **(c–d)** under visible light irradiation by: **(c)** TIPS and **(d)** ES membranes.

Table S1. Reaction rate (k_{app}) for composite membranes by photocatalysis under UV irradiation.

	k_{app} (min^{-1})									
	HFP					TrFE				
	TiO_2	7T-3Z	5T-5Z	3T-7Z	ZnO	TiO_2	7T-3Z	5T-5Z	3T-7Z	ZnO
**TIPS**	0.0001	0.0005	0.0007	0.0006	0.0009	0.0018	0.0007	0.0009	0.0012	0.0015
**ES**	0.0018	0.0007	0.0009	0.0011	0.0015	0.0005	0.0008	0.0010	0.0026	0.0020

Table S2. Reaction rate (kapp) for composite membranes by photocatalysis under visible light irradiation.

	k_{app} (min^{-1})									
	HFP					TrFE				
	TiO_2	7T-3Z	5T-5Z	3T-7Z	ZnO	TiO_2	7T-3Z	5T-5Z	3T-7Z	ZnO
**TIPS**	0.0005	0.0008	0.0010	0.0013	0.0005	0.0003	0.0003	0.0005	0.0007	0.0005
**ES**	0.0004	0.0008	0.0019	0.0019	0.0017	0.0004	0.0010	0.0016	0.0016	0.0012

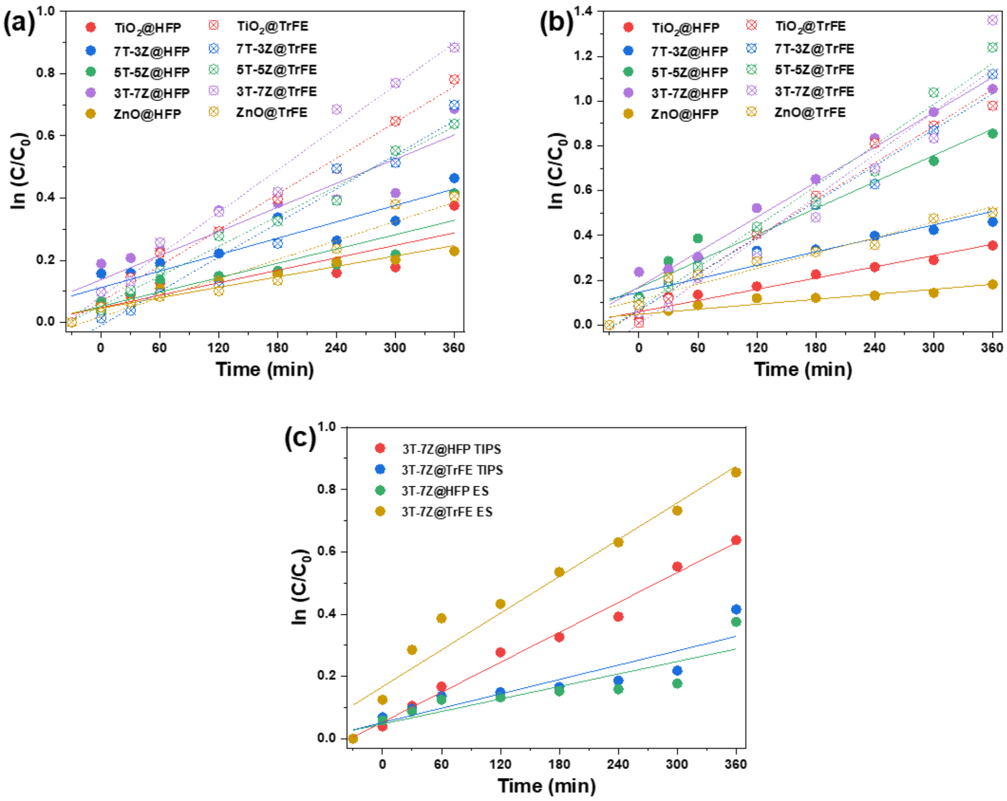

Figure S3. Degradation rate for the photocatalytic CIP degradation **(a–b)** under UV irradiation and **(c–d)** under visible light irradiation by the top-performance 3T-7Z@-based membrane.

Table S3. Reaction rate (k_{app}) for composite membranes by sonophotocatalysis under UV irradiation.

	k_{app} (min^{-1})									
	HFP					TrFE				
	TiO_2	7T-3Z	5T-5Z	3T-7Z	ZnO	TiO_2	7T-3Z	5T-5Z	3T-7Z	ZnO
TIPS	0.0007	0.0009	0.0008	0.0013	0.0006	0.0019	0.0018	0.0016	0.0023	0.0010
ES	0.0008	0.0010	0.0020	0.0026	0.0004	0.0028	0.0027	0.0031	0.0032	0.0012

Table S4. Reaction rate (k_{app}) for composite membranes by sonophotocatalysis under visible light irradiation.

	k_{app} (min^{-1})	
	3T-7Z	
	HFP	TrFE
TIPS	0.0016	0.0007
ES	0.0008	0.0020

Table S5. Degradation rates under PC and SPC, and calculated REF for the 3T-7Z-based composite membranes.

Polymer	Method	UV			VIS		
		Rate (min^{-1})		REF	Rate (min^{-1})		REF
		PC	SPC		PC	SPC	
PVDF-HFP	TIPS	0.0006	0.0013	2.17	0.0013	0.0016	1.23
	ES	0.0011	0.0026	2.36	0.0019	0.0008	0.42
PVDF-TrFE	TIPS	0.0012	0.0023	1.92	0.0007	0.0007	1.00
	ES	0.0026	0.0032	1.23	0.0016	0.0020	1.25
